# Infectious Disease in the Workplace: Quantifying Uncertainty in Transmission

**DOI:** 10.1007/s11538-023-01249-x

**Published:** 2024-02-01

**Authors:** Jonathan I. D. Hamley, Guido Beldi, Daniel Sánchez-Taltavull

**Affiliations:** 1 Department of Visceral Surgery and Medicine, Inselspital, Bern University Hospital, University of Bern, Bern, Switzerland; 2 Multidisciplinary Center for Infectious Diseases, University of Bern, Bern, Switzerland; 3 Bern Center for Precision Medicine, Bern, Switzerland

**Keywords:** Epidemiology, Stochastic modelling, Uncertainty, Coefficient of variation, Multiscale modelling

## Abstract

Understanding disease transmission in the workplace is essential for protecting workers. To model disease outbreaks, the small populations in many workplaces require that stochastic effects are considered, which results in higher uncertainty. The aim of this study was to quantify and interpret the uncertainty inherent in such circumstances. We assessed how uncertainty of an outbreak in workplaces depends on i) the infection dynamics in the community, ii) the workforce size, iii) spatial structure in the workplace, iv) heterogeneity in susceptibility of workers, and v) heterogeneity in infectiousness of workers. To address these questions, we developed a multiscale model: A deterministic model to predict community transmission, and a stochastic model to predict workplace transmission. We extended this basic workplace model to allow for spatial structure, and heterogeneity in susceptibility and infectiousness in workers. We found a non-monotonic relationship between the workplace transmission rate and the coefficient of variation (CV), which we use as a measure of uncertainty. Increasing community transmission, workforce size and heterogeneity in susceptibility decreased the CV. Conversely, increasing the level of spatial structure and heterogeneity in infectiousness increased the CV. However, when the model predicts bimodal distributions, for example when community transmission is low and workplace transmission is high, the CV fails to capture this uncertainty. Overall, our work informs modellers and policy makers on how model complexity impacts outbreak uncertainty. In particular: workforce size, community and workplace transmission, spatial structure and individual heterogeneity contribute in a specific and individual manner to the predicted workplace outbreak size distribution.

**Supplementary Information:**

The online version contains supplementary material available at 10.1007/s11538-023-01249-x.

## Introduction

Predicting infections in a workforce is essential for tailoring preventative interventions, maintaining productivity, and prioritising vaccinations. This has led to many mathematical models for workplace transmission (Lloyd-Smith et al. 2003; Hill et al. 2021; Evans et al. 2021; Jarvis and Kelley 2021; Sánchez-Taltavull et al. 2021; Sanchez-Taltavull et al. 2021). The strength of workplace disease transmission for respiratory infections, such as influenza and SARS-CoV-2 differs between occupations (Lietz et al. 2016; Eisen 2020; Mutambudzi et al. 2021; Murti et al. 2021; Chen et al. 2021). Two common characteristics of workplace transmission models are i) they simultaneously consider the epidemiological dynamics in the wider community, i.e. outside the workplace, and those within the workplace, and ii) they account for the small population sizes typically found in workplaces. For small population sizes, stochastic effects are more pronounced than in larger ones, making deterministic models inappropriate.

In stochastic models there are two outputs of interest. The first is the average of the stochastic runs of the model, and the second is the distribution of these stochastic runs. In some areas of a model’s parameter space, the average outbreak size is not an accurate representation of the stochastic realisations, which can lead to different outbreak size distributions (Bailey 1950, 1964; Keeling and Ross 2008). For example, epidemiological SIR models can predict bimodal distributions for some population sizes and transmission rates (Bailey 1953). A bimodal distribution of outbreak sizes indicates particularly high uncertainty, since it predicts an equal probability of a large and small outbreak. The shape of the outbreak size distribution predicted by a stochastic model can be summarised by the coefficient of variation (Drake 2006), which is a measure of uncertainty of the predicted outbreak size and is defined as the mean divided by the standard deviation. For example, when the outbreak size distribution is highly overdispersed (i.e. it has a long tail), the coefficient of variation is large. However, the coefficient of variation does not give information on the exact shape of the distribution.

Increasing the complexity of epidemiological models can change their behaviour, such as the addition of individual- or population-level heterogeneity. Spatial structure is an example of population-level heterogeneity and has been studied in Ball and Lyne (2001), Ball and Neal (2002). Individual-level heterogeneity can be accounted for by adding variation in infectiousness (Lloyd-Smith et al. 2005; Garske and Rhodes 2008; Hartfield and Alizon 2013) or by adding variation in susceptibility (Gomes et al. 2021, 2022). Additional model complexity can increase the accuracy of the model, and the number of scenarios it can be applied to. However, reducing complexity allows increased tractability of the models and reduces the number of parameters to be estimated from data.

In deterministic models, there are clears costs and benefits of increasing model complexity (Bussell et al. 2019). In stochastic models, increasing complexity may influence not only the average model behaviour, but also the variation across individual realisations of the model. Investigating the complexity of stochastic epidemiological models is important in the context of outbreak size uncertainty. Changes in the complexity of the models can influence how representative the average behaviour is of the stochastic dynamics. Thus, our understanding of how complexity determines predictions of deterministic models is not always applicable to stochastic models. Models for public health-related questions are becoming increasingly complex. Therefore, it is essential to understand when increasing model complexity is necessary (Pellis et al. 2020).

The predictions of workplace models depend on community and workplace transmission. For example, it has been observed that when community transmission is low and workplace transmission is high, there is a low probability of a large workplace outbreak (Sánchez-Taltavull et al. 2021). This could lead to a bimodal outbreak size distribution, and therefore high uncertainty. Our goal, is to investigate for what levels of model complexity stochastic effects are dominant, and therefore when additional model complexity needs to be included for predicting outbreak uncertainty in workplaces.

Our aim is to understand how predicted uncertainty in workplace disease outbreaks depends on model complexity. To this end, we develop a stochastic individual-based epidemiological model for workplace transmission and combine it with a community transmission model. We determine how workplace outbreak uncertainty depends on the strength of workplace transmission and (i) the dynamics in the wider community, (ii) the workforce size, (iii) spatial structure in the workplace, (iv) heterogeneity in susceptibility between workers, (v) heterogeneity in infectiousness between workers. We use the coefficient of variation as a measure of outbreak uncertainty, and identify when this measure does not match the information from the entire outbreak size distribution. The intended impact of this work is that it will inform future model development for workplace disease dynamics by identifying when increasing model complexity influences uncertainty due to stochastic effects.

## Methods and Models

### Mathematical Models

To simulate infections in the community outside of the workplace, we used a population-based deterministic model, as the population is assumed to be sufficiently large that stochastic effects can be ignored. Community variables are denoted with subscript *C*. To simulate workplace transmission we developed a stochastic individual-based model in which variables are denoted with subscript *W*.

#### Community Transmission Model

A system of ordinary differential equations based on a widely used framework for representing epidemiological dynamics (Anderson and May 1992) accounts for the number of susceptible ($S_C$), exposed ($E_C$), infected ($I_C$), hospitalised ($H_C$), recovered ($R_C$) and dead ($D_C$) individuals at time *t*:

1 $$\begin{aligned} \frac{dS_C}{dt}\ {}= & {} -\alpha (t) {S_{C}I_{C}}, \end{aligned}$$

2 $$\begin{aligned} \frac{dE_C}{dt}\ {}= & {} \alpha (t) {S_{C}I_{C}-lE_{C}},\end{aligned}$$

3 $$\begin{aligned} \frac{dI_C}{dt}\ {}= & {} {lE_{C}-rI_{C}(1-\epsilon _{1})-h\epsilon _{1}I_{C}},\end{aligned}$$

4 $$\begin{aligned} \frac{dH_C}{dt}\ {}= & {} {h\epsilon _{1}I_{C}}-wH_{C}(1-\epsilon _{2}) - dH_{C}\epsilon _{2},\end{aligned}$$

5 $$\begin{aligned} \frac{dR_C}{dt}\ {}= & {} {rI_{C}(1-\epsilon _{1})+wH_{C}(1-\epsilon _{2})},\end{aligned}$$

6 $$\begin{aligned} \frac{dD_C}{dt}\ {}= & {} dH_{C}\epsilon _{2} \end{aligned}$$

where $\alpha (t)$ is the infection rate, 1/*l* is the mean latency period, 1/*r* is the mean infection duration, *h* is the hospitalisation rate, $\epsilon _1$ is probability of hospitalisation, *w* is the recovery rate of hospitalised patients and $\epsilon _2$ is the probability of death for hospitalised patients. The parameter descriptions are shown in Table 1. Although we do not use them in our study, we account for hospitalisations and deaths since these can allow calibration of the model to data if required. At *t* = 0, we assume $S_C$ = $N_C - 1$, $I_C$ = 1 and all other variables are 0. Following Althaus et al. (2020) we represented the decline in community transmission due to government implemented non-pharmaceutical interventions to reduce community transmission using:

7 $$\begin{aligned} \alpha (t) = \frac{\pi }{N_{C}}\left( 1-\frac{1 - \kappa }{1 + e^{-\nu (t - \tau )}}\right) \end{aligned}$$

where $\alpha (t)$ is the infection rate in the community at time *t* and $N_C$ is the community size. The values for $\kappa$, $\nu$ and $\tau$ remain fixed and we vary $\pi$ to study different community infection dynamics.

#### Basic Workplace Transmission Model

*Transitions Between Classes* To model transmission within the workplace we developed a stochastic individual-based model [based on the stochastic model used in Sanchez-Taltavull et al. (2021)] which tracks the infection status of each individual, which is denoted by subscript *i*. At *t* = 0, we assume there are no infections in the workplace. The probabilities used for the transition events were converted from the rates described in Table 1, using $p = 1- e^{-a dt}$, where *p* is the probability, *a* is the rate and *dt* is the time step. Changes in the infection status of an individual are defined by stochastic transition events, whether a change in status occurs or not is drawn from a binomial distribution. Each individual, *i*, can be in one of 5 states at time *t*: $S_W(i)$ (susceptible), $E_W(i)$ (exposed), $I_W(i)$ (infected, symptomatic), $A_W(i)$ (infected, asymptomatic) or $R_W(i)$ (recovered). When referring to the total number of individuals in a class we omit subscript *i*. The total population size is represented as $N_W$. Here we describe the transitions between the classes, an algorithm for implementing the model computationally can be found in the supplementary information.

8 $$\begin{aligned}{} & {} S_{W(i)}(t) \rightarrow S_{W(i)}(t + dt) - 1 \end{aligned}$$

9 $$\begin{aligned}{} & {} E_{W(i)}(t) \rightarrow E_{W(i)}(t + dt) + 1 \end{aligned}$$

with probability $$1- e^{-S_{W(i)}(\beta \frac{A_{W}}{N_{W}} + \alpha I_{C}) dt}$$

Note that the transition from susceptible to exposed in the work place depends on the number of infections in the workplace as well as those in the community. The equations above assume that transmission depends on the frequency of infected individuals in the workplace. Alternatively, we can assume that individuals interact more as the workforce size increases, giving density-dependent transmission:

10 $$\begin{aligned}{} & {} S_{W(i)}(t) \rightarrow S_{W(i)}(t + dt) - 1 \end{aligned}$$

11 $$\begin{aligned}{} & {} E_{W(i)}(t) \rightarrow E_{W(i)}(t + dt) + 1 \end{aligned}$$

with probability $$1- e^{-S_{W(i)}(\beta A_{W} + \alpha I_{C}) dt}$$

Note that we assume frequency-dependent transmission throughout unless otherwise stated. We assume that when a worker develops symptoms (they are in class $I_{W}$) they quarantine. Latent individuals become symptomatic ($I_{W(i)}$) or asymptomatic ($A_{W(i)}$) following:

12 $$\begin{aligned} E_{W(i)}(t) \rightarrow E_{W(i)}(t + dt) - 1\end{aligned}$$

with probability $$1- e^{-E_{W(i)}(t)l dt}$$

13 $$\begin{aligned} I_{W(i)}(t) \rightarrow I_{W(i)}(t + dt) + 1\end{aligned}$$

with probability $$\epsilon _{3} (1- e^{-E_{W(i)}(t)l dt})$$

14 $$\begin{aligned} A_{W(i)}(t) \rightarrow A_{W(i)}(t + dt) + 1\end{aligned}$$

with probability $$(1-\epsilon _{3})(1- e^{-E_{W(i)}(t)l dt})$$

Both symptomatic and asymptomatic individuals recover at rate *r*

15 $$\begin{aligned}{} & {} I_{W(i)}(t) \rightarrow I_{W(i)}(t + dt) - 1 \end{aligned}$$

16 $$\begin{aligned}{} & {} R_{W(i)}(t) \rightarrow R_{W(i)}(t + dt) + 1 \end{aligned}$$

with probability $$1- e^{-I_{W(i)}(t)r dt}$$

17 $$\begin{aligned}{} & {} A_{W(i)}(t) \rightarrow A_{W(i)}(t + dt) - 1 \end{aligned}$$

18 $$\begin{aligned}{} & {} R_{W(i)}(t) \rightarrow R_{W(i)}(t + dt) + 1 \end{aligned}$$

with probability $$1- e^{-A_{W(i)}(t)r dt}$$

The mean-field dynamics are shown in the supplementary information.

### Scenarios and Model Extensions

Using the basic model formulation described in equations 1 to 18, we perform a sensitivity analysis of various parameters to study their impact on the epidemiological dynamics and outbreak size distributions. We also make extensions to our basic framework and investigate their impact on the outbreak size distribution (described in the sections below). For each parameter variation or model extension we vary $\beta$ between 0 and 2.5 to study the impact of workplace transmission on the epidemiological dynamics in the workplace. We define the outbreak size as the proportion of recovered individuals at the final time step (*t* = 110 days). We calculated the mean outbreak size (over repeat simulations) for each parameter set, as well as the coefficient of variation ($CV = \frac{standard\,deviation}{mean}$) as a measure of outbreak uncertainty. For transmission in the community, we fixed all parameters at values applicable to the early stages of the SARS-CoV-2 outbreak (Table 1), apart from $\pi$, which was varied to alter community transmission dynamics. Note although we use some parameter values specific to SARS-CoV-2 (mean latency period (1/*l*), mean infection duration (1/*r*), hospitalisation rate (*h*), probability of hospitalisation ($\epsilon _1$), recovery rate of infected individuals (*w*), probability of death for hospitalised patients ($\epsilon _2$)) (see Sanchez-Taltavull et al. 2021), our objective is to demonstrate the qualitative behaviour of the models rather than to produce predictions for a specific respiratory virus.

To solve the ordinary differential equations for city transmission (Eqs. 1–5) we used the ‘deSolve’ package (Soetaert et al. 2010) in R version 4 (R Core Team 2020), using *dt* = 4/24 days. The algorithm for simulating the stochastic, individual-based, workplace transmission model can be found in supplementary information. To calculate transitions between states (i.e. a susceptible individual moving to the exposed class) we used the *rbinom* function (R Core Team 2020). We conducted 5000 stochastic model runs for each parameter set with the exception of the spatial simulations (described below, 1000 stochastic model runs).

#### Assessment of How Community Transmission Determines the Outbreak Size Distribution

To study the impact of community transmission on the epidemiological dynamics in the workplace, we vary parameter $\pi$ between 0.48 and 1.12, which is found in equation 7. Increasing $\pi$ results in a higher number of individuals being infected during the outbreak.

#### Assessment of How the Workforce Size Determines the Outbreak Size Distribution

To study the impact of workforce size on the epidemiological dynamics in the workplace, we varied the workforce size ($N_W$) between 20 and 1000 workers.

#### Assessment of How Spatial Structure in the Workplace Determines the Outbreak Size Distribution

We account for spatial structure by assuming the workplace is divided into *G* subgroups, where the within-group transmission rate is given by $\beta _{g}$ and the between-group transmission rate is given by $\beta$. Subscript *g* denotes the group an individual belongs to (e.g $S_{W(g, i)}$ denotes individual *i* who is susceptible and in group *g*) or a group within a class (e.g $A_{W(g)}$ denotes all the individuals in class $A_W$). The workforce is divided into equally sized groups, but variations of this assumption are shown in the Supplementary Information. We assume that transmission within a group is a function of the proportion of infected individuals in that group, whereas transmission from outside an individual’s groups is a function of the proportion of infected individuals in all other groups.

19 $$\begin{aligned}{} & {} S_{W(g, i)}(t) \rightarrow S_{W(g, i)}(t + dt) - 1 \end{aligned}$$

20 $$\begin{aligned}{} & {} E_{W(g, i)}(t) \rightarrow E_{W(g, i)}(t + dt) + 1 \end{aligned}$$

with probability $$1- e^{-S_{W(g, i)}\left( \beta _{g} \frac{A_{W(g)}}{N_{W(g)}} + {\alpha I_{C}} + \beta \frac{A_{W} - A_{W(g)}}{N_{W} - N_{W(g)}}\right) dt}$$

#### Assessment of How Individual-Level Heterogeneity in Infectiousness and Susceptibility Determines the Outbreak Size Distribution

We consider two types of individual heterogeneity; infectiousness per unit time and susceptibility. To investigate the impact of different levels of overdispersion in infectiousness and susceptibility on the predicted outbreak size distribution, we assumed a gamma distribution for each trait. We use the form of the gamma distribution parameterised by the shape (*k*) and rate ($\omega$):

21 $$\begin{aligned} f(x) = \frac{\omega ^{k}}{\Gamma (k)} {x^{k-1}e^{-\omega x}}\end{aligned}$$

The impact of varying *k* and $\omega$ is shown in the Supplementary Information (Fig. S1). We assume always that $k = \omega$, such that the mean of the distribution is 1. By doing this, the distribution can then be multiplied by the epidemiological parameter of interest to make the mean of the distribution take this value. We use subscripts to distinguish between *k* and $\omega$ for the different epidemiological parameters:

22 $$\begin{aligned}{} & {} \beta _{i} = gamma(k_{\rho }, \omega _{\rho })\beta \end{aligned}$$

23 $$\begin{aligned}{} & {} \phi _{i} = gamma(k_{z}, \omega _{z}) \end{aligned}$$

where $\beta _{i}$ is the infectiousness and $\phi _{i}$ is the susceptibility. Subscripts $\rho$ and *z* indicate the parameters used for the infectiousness and susceptibility distributions, respectively. Note that the mean susceptibility in the population is assumed to be unity, and we allow heterogeneity in either susceptibility, infectiousness or both. We calculate the probability of the transition of individual *i*, from $S_W(i)$ to $E_W(i)$ accounting for the mean infectiousness in the population (${\bar{\beta }} = \frac{\sum _{i=1}^{N_{W}} \beta _{i}}{A_{W}}$, assuming $\beta _{i}$ = 0 for all individuals not in the asymptomatic class), and the individual’s level of susceptibility ($\phi _{i}$):

24 $$\begin{aligned}{} & {} S_{W(i)}(t) \rightarrow S_{W(i)}(t + dt) - 1 \end{aligned}$$

25 $$\begin{aligned}{} & {} E_{W(i)}(t) \rightarrow E_{W(i)}(t + dt) + 1 \end{aligned}$$

with probability $$1- e^{-S_{W(i)}\phi _{i}({\bar{\beta }} \frac{A_{W}}{N_{W}} + {\alpha I_{C}}) dt}$$

We used the *rgamma* function (R Core Team 2020) to draw values for individual-level infectiousness and susceptibility. Note that since all individuals mix homogeneously, the term that appears as the transmission rate is the mean of the individual levels of infectiousness. When assuming there is no heterogeneity in infectiousness or susceptibility, $\beta$ and $\phi$ take the following constant values:

26 $$\begin{aligned}{} & {} \beta _{i} = \beta \end{aligned}$$

27 $$\begin{aligned}{} & {} \phi _{i} = 1 \end{aligned}$$

**Table 1:** Parameter descriptions for both the community and workplace models

Parameter	Value	Description
$\beta$	0–2.5 $\hbox {days}^{-1}$	Workplace transmission
${{\bar{\beta }}}$	0–1.5 $\hbox {days}^{-1}$	Mean workplace transmission rate when assuming heterogeneity in infectiousness
$\beta _{g}$	0.5–0.9 $\hbox {days}^{-1}$	Within-group transmission rate
1/*l*	5.2 days	Mean latency period
*r*	1/6 $\hbox {days}^{-1}$	Recovery rate
*w*	1/10 $\hbox {days}^{-1}$	Recovery rate for hospitalised individuals
*h*	1/6 $\hbox {days}^{-1}$	Hospitalisation rate
*d*	1/10 $\hbox {days}^{-1}$	Death rate
$\pi$	0.48–1.12 $\hbox {days}^{-1}$	Infection rate in the community
$\kappa$	0.016	Relative transmission in the community after NPIs
$\nu$	0.3	Slope of the sigmoid function
$\tau$	50	Midpoint of transmission reduction
$N_{C}$	1000000	Community population size
$N_{W}$	20–1000	Workplace population size
$\omega _\rho$	0–3	Gamma rate, infectiousness
$k_\rho$	0–3	Gamma shape, infectiousness
$\omega _z$	0–3	Gamma rate, susceptibility
$k_z$	0–3	Gamma shape, susceptibility
$\epsilon _{1}$	0.025	Probability of being hospitalised
$\epsilon _{2}$	0.37	Probability of death
$\epsilon _{3}$	0.3	Probability of being symptomatic

## Results

### Levels of Transmission in the City and the Workplace Influence Uncertainty

**Fig. 2:**
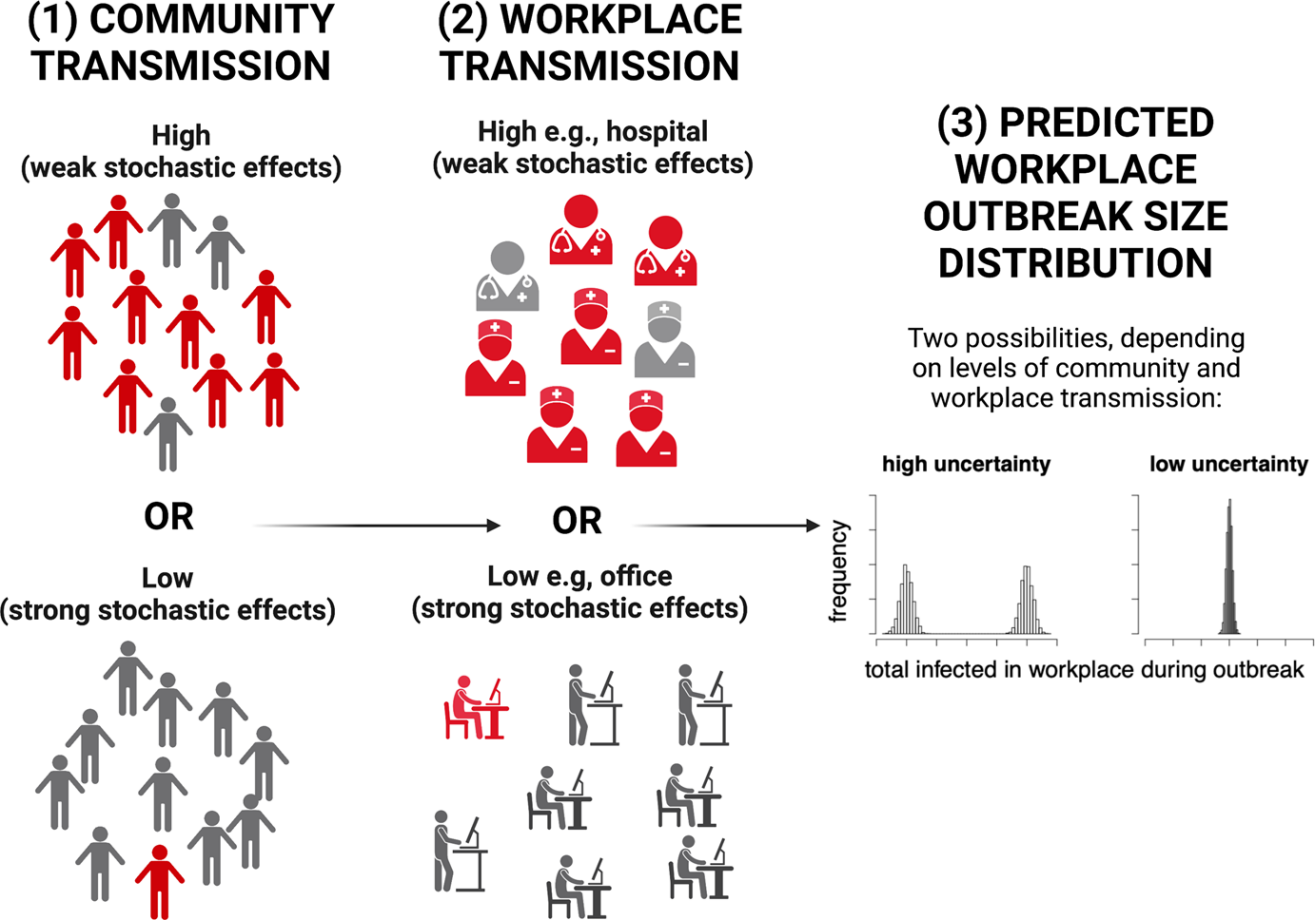
Uncertainty in the number of infected workers depends on stochastic effects, which are dependent on the strength of both community and within-workplace transmission. Different combinations of workplace and community transmission can lead to different workplace outbreak size distributions. Created with BioRender.com

We first investigated the interaction between community and workplace transmission on the outbreak size distribution (Fig. 1 depicts the approach). When assuming no individual or spatial heterogeneity, increasing workplace and community transmission increased the mean outbreak size (Fig. 2). However, outbreak size distributions strongly depend on the combination of workplace and community transmission. Low community and workplace transmission led to a right skewed distribution (Fig. 2a) whereas high community transmission with high workplace transmission led to left skewed distribution (Fig. 2b). Low workplace and high community transmission led to a Gaussian distribution (Fig. 2c). Low community transmission combined with high workplace transmission produce a bimodal distribution of outbreak size (Fig. 2d). Therefore, we see that different combinations of community and workplace transmission produce different outbreak size distributions.

Increasing the level of community transmission increased the mean workplace outbreak size (Fig. 2e and f). We found non-monotonic changes in the coefficient of variation as workplace transmission ($\beta$) increased. Namely, the coefficient of variation increases for low values of $\beta$, until it peaks and then decreases monotonically for larger values of $\beta$. This was most pronounced for lower levels of community transmission (Fig. 2g).

**Fig. 3:**
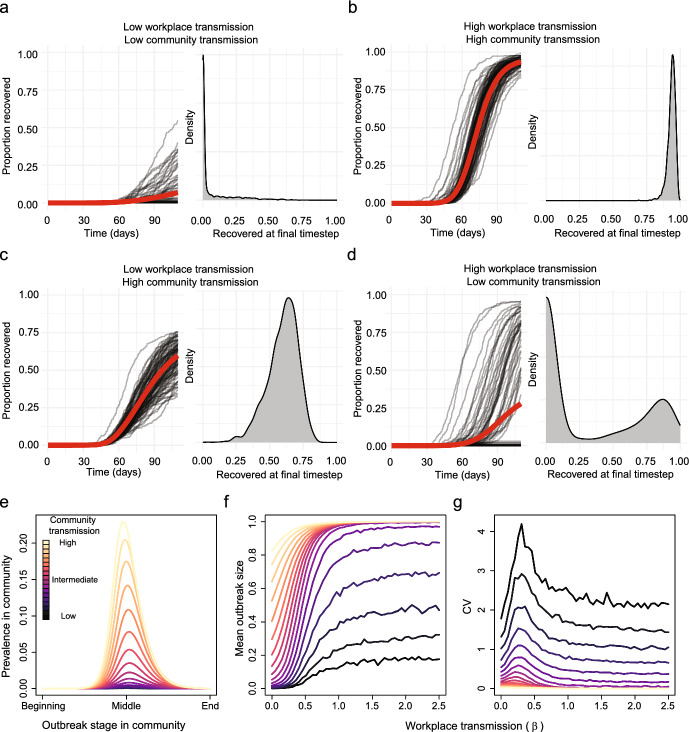
**a**–**d** The temporal dynamics given by the mean (red) and individual stochastic runs (black) for recovered individuals and the distribution of final outbreak sizes for various strengths of within-workplace ($\beta$) and community transmission assuming no individual or spatial heterogeneity. Each panel, comprising of the time series (left) and outbreak size distribution (right), corresponds to one combination of the strengths of transmission in the workplace and wider community shown in Fig. 1**e** The community dynamics for the simulations shown in panels **f** & **g**, $\pi$ is varied between 0.48 and 1.12. **f** The mean outbreak size (prevalence) for different levels of workplace and community transmission. **g** The coefficient of variation for different levels of workplace and community transmission (Color figure online)

**Fig. 4:**
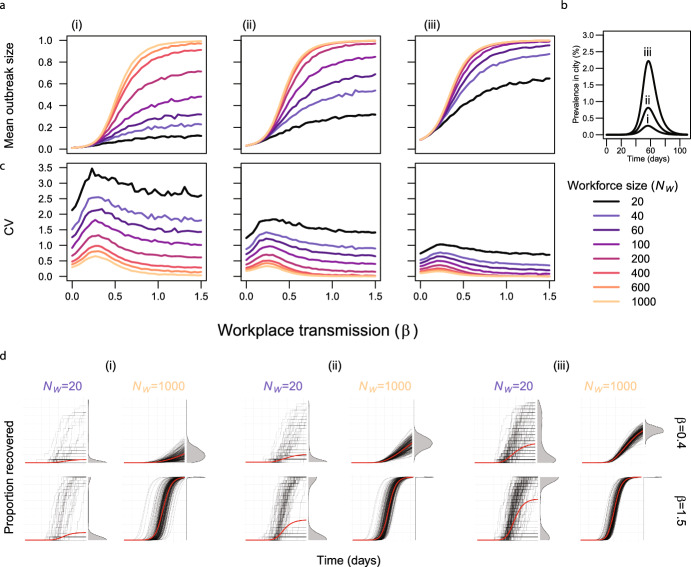
**a** The outbreak size (the mean proportion of recovered individuals at the end of the outbreak) for different workforce sizes and strengths of community (i-iii) and workplace transmission. **b** The community infection dynamics assumed in panels **a**, **c** & **d**. **c** The coefficient of variation of the outbreak size in the workplace. d The temporal trajectories of the proportion of recovered individuals for different combinations of workforce size ($N_W$) and workplace transmission ($\beta$) for three strengths of community transmission (Color figure online)

### The Impact of Workforce Size on Uncertainty Depends on Community and Workplace Transmission

Larger work forces were generally associated with larger mean outbreak sizes (Fig. 3a i-iii). As community transmission increases, the mean outbreak size increases. The assumptions for the community dynamics are shown in Fig. 3b. The coefficient of variation shows a non-monotonous dependence on $\beta$ (Fig. 3c i-iii). For large workforce sizes, the coefficient of variation decreases, and the non-monotonous behaviour becomes less pronounced. The coefficient of variation decreases as community transmission increases in a similar way to increasing workforce size (e.g compare Fig. 3c i with Fig. 3c iii). For low community transmission (Fig. 3d i) and low workplace transmission ($\beta =0.4$), most of the simulations remain at 0 for a small workforce size ($N_w=20$). For a high workforce size ($N_W=1000$), all the simulations fluctuate around a low mean (Fig. 3d i). However, for large values of workplace transmission ($\beta =1.5$) we observe a bimodal distribution for a small workforce size, which becomes more pronounced as community transmission increases (Fig. 3d i-iii). This uncertainty is not reflected in the coefficient of variation which is predicted to be lower for the bimodal case (compare Fig 3d ii, the top left panel with the bottom left panel, and Fig 3d iii, the top left panel with the bottom left panel). Increasing the strength of community transmission (Fig. 3d i-iii) does not qualitatively change the impact of workforce size and workplace transmission on the outbreak size distributions.

For frequency-dependent transmission, a similar value of workplace transmission gave the highest coefficient of variation for each workforce size for a given level of community transmission (Fig. 3). We found that switching from frequency-dependent to density-dependent transmission in the workplace influenced which value of workplace transmission produced the highest coefficient of variation across the different workforce sizes (Supplementary Information, Fig. S2).

**Fig. 5:**
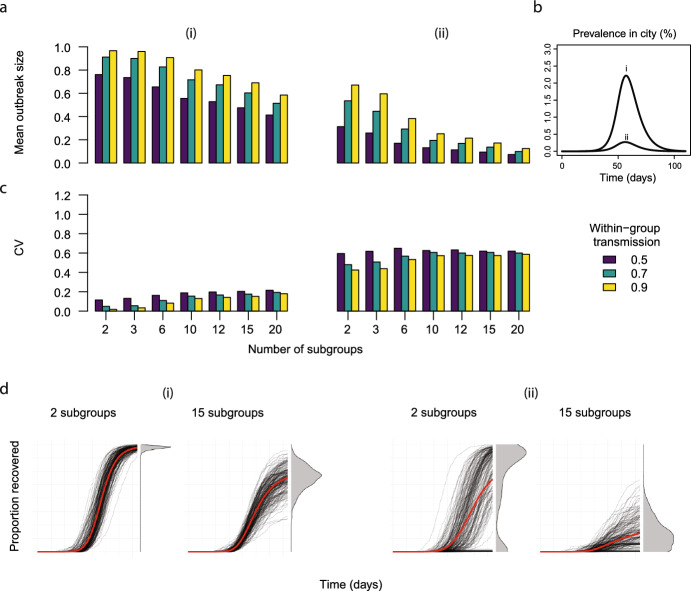
a The mean outbreak sizes for different numbers of within-workplace subgroups and strengths of within-subgroup and community transmission (i-ii) for between group transmission ($\beta$) = 0.02. b The assumed community transmission dynamics in panels a and c. c The coefficient of variation for outbreak size for different numbers of within-workplace subgroups and strengths of within-subgroup and community transmission (i-ii). d The individual stochastic (black) and mean (red) trajectories for two levels of spatial heterogeneity(2 and 15 subgroups). Workforce size ($N_W$) = 300, transmission within groups ($\beta _g$) = 0.9 (Color figure online)

### Increasing Spatial Heterogeneity can Increase Uncertainty Depending on Community Transmission

In addition to the effects of smaller workforce sizes that we characterised in Sect. 3.2, we aimed to understand the impact of spatial structure on the mean outbreak size and on the coefficient of variation. Decreasing the number of closely interacting groups in the workplace increases the mean outbreak size for high and low levels of community transmission (Fig. 4a i-ii and b). Similarly, increasing the within-group transmission rate increases the mean outbreak size (Fig. 4a i-ii, compare the purple, green and yellow bars). The strength of community transmission regulates the impact of spatial structure on outbreak size uncertainty. Namely, when community transmission is high, decreasing the number of workplace subgroups decreases the coefficient of variation (Fig. 4c i). When community transmission is low, decreasing the number of workplace groups has no impact on the coefficient of variation when the within-group transmission rate = 0.5. When considering the outbreak size distributions for high community transmission (Fig. 4d i), we observe that uncertainty behaves as described by the coefficient of variation. However for low community transmission, the coefficient of variation does not capture the differences in the outbreak size distributions (Fig 4d ii). The distribution is bimodal when there are 2 subgroups and overdispersed when there are 15, but the coefficient of variation does not increase in the bimodal case. A description of the impact of varying group sizes can be found in the supplementary information (Figs. S3–5).

**Fig. 6:**
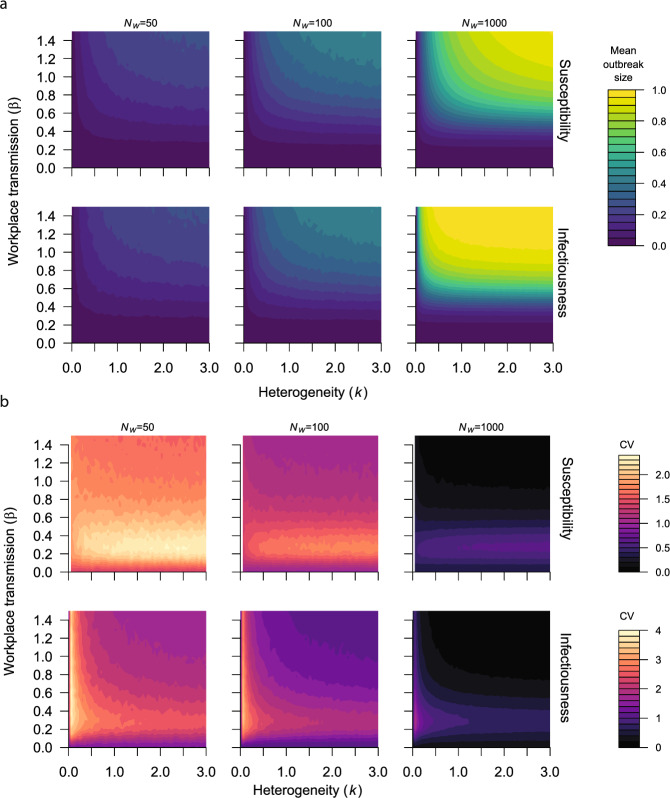
**a** Mean outbreak sizes for different levels of heterogeneity (*k*) in susceptibility and infectiousness for various workforce sizes ($N_W$ = 50, 100, 1000). **b** The coefficient of variation for outbreak size for different levels of heterogeneity (*k*) in susceptibility and infectiousness for various workforce sizes ($N_W$ = 50, 100, 1000). Note, there are two scales in panel **b**. Higher values of *k* give lower heterogeneity (Color figure online)

### Heterogeneity in Infectiousness and Susceptibility Differ in their Impact on Outbreak Uncertainty

Our next step is to identify how heterogeneity in susceptibility and infectiousness influences the mean outbreak size and the coefficient of variation. For both infectiousness and susceptibility, increasing heterogeneity (i.e. lower values of *k*) reduced the mean outbreak size. Additionally, increasing the workforce size increased the mean outbreak size for both assumptions (Fig. 5a).

**Fig. 7:**
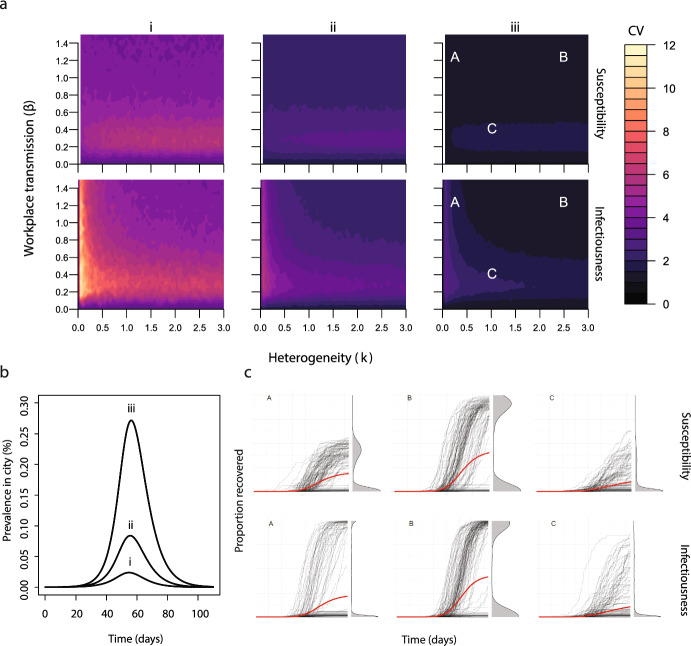
**a** The coefficient of variation for outbreak size for different levels of heterogeneity in susceptibility (top row) and infectiousness (bottom row) for different values of workplace transmission ($\beta$). **b** The assumed community infection dynamics in panels a and c. c The individual stochastic (black) and mean (red) temporal trajectories of the prevalence of recovered individuals for different combinations of workplace transmission ($\beta$) and heterogeneity (*k*) in susceptibility (top row) and infectiousness (bottom row), for high community transmission (line iii in panel b) (Color figure online)

As the workforce size increases, the coefficient of variation decreases for both cases. However, we observe qualitatively different behaviour between susceptibility and infectiousness. Namely, for susceptibility, high values of *k*, and low values of workplace transmission can result in a high coefficient of variation (Fig. 5b, top row). While, for infectiousness low values of *k* (corresponding to high heterogeneity) result in a high coefficient of variation (Fig. 5b, bottom row). The coefficient of variation decreased with the strength of community transmission for both infectiousness and susceptibility (Fig. 6a and b). However, the qualitative behavior of the coefficient of variation, as a function of heterogeneity and workplace transmission, did not change as community transmission increased. This is similar to the impact of increasing the workforce size (Figs. 5b and 6a). The coefficient of variation does not capture the behaviour shown in the the individual stochastic trajectories (Fig. 6c). For example, the parameter combinations at point C produce a higher coefficient of variation than those at point B, but the individual trajectories indicated by points B show bimodal outbreak size distributions. When assuming heterogeneity in both infectiousness and susceptibility and low transmission in the workplace, increasing levels of heterogeneity had a similar impact on the coefficient of variation to when each assumption was tested separately (Supplementary Information, Fig. S6). For a given level of heterogeneity in infectiousness, increasing heterogeneity in susceptibility decreased the coefficient or variation. For a given level of heterogeneity in susceptibility, increasing heterogeneity increased the coefficient of variation. When workplace transmission was high, increasing heterogeneity in infectiousness, but not susceptibility, impacted the coefficient of variation. A summary of the main findings of this paper is shown in Table 2.

**Table 2:** Summary of the main findings

$\uparrow$ in parameter	Impact on the workplace outbreak size distribution	Figure reference
Community transmission	$\uparrow$ mean $\downarrow$ CV	2, 3
Workplace transmission	$\uparrow$ mean, non-monotonic changes in CV	2, 3
Workforce size	$\uparrow$ mean $\downarrow$ CV	3
Number of workplace groups	$\downarrow$ mean $\uparrow$ CV	4
Heterogeneity in infectiousness	$\downarrow$ mean $\uparrow$ CV	5, 6
Heterogeneity in susceptibility	$\downarrow$ mean $\downarrow$ CV	5, 6

## Discussion

An underlying goal of theoretical epidemiology is to account for sufficient biological detail to provide accurate predictions whilst maintaining model tractability. In small populations (e.g., workplaces) stochastic effects determine uncertainty in model predictions. We have studied how varying the level of complexity in stochastic models determines uncertainty in outbreak size in a workplace. The utility of this work is two-fold; First, it identifies biological aspects (e.g., heterogeneity in infectiousness) which might influence the shape of the outbreak size distribution. Second, it demonstrates which levels of model complexity are required to predict stochastic uncertainty for different transmission conditions. Previous work has used the coefficient of variation as a measure of outbreak uncertainty (Drake 2006) and examined outbreak size distributions (Bailey 1953). Other work has considered the impact of infection duration distributions of outbreak probabilities (Britton and Lindenstrand 2009). We extend their work by assessing the performance of the coefficient of variation as a measure of uncertainty for various levels of model complexity. Additionally, we compare the impact of different model assumption on the coefficient of variation. Our findings have the potential to inform the development of future models for workplace (or school and nursing home) disease transmission by informing what level of biological detail is required.

### The Coefficient of Variation can be a Misleading Measure of Uncertainty when Community Transmission is Low or the Workforce is Small

For all the assumptions tested, a straight forward finding was that increasing biological detail is more important when community transmission is low. When there is strong transmission in the wider community, workers become infected even when workplace transmission is low, and therefore any stochastic effects associated with workplace transmission have little impact on model behaviour. Thus, as community transmission increases, adding additional biological details to workplace models does not necessarily increase the accuracy of predicted uncertainty. For low levels of community transmission, increasing workplace transmission led to a decrease in the coefficient of variation. However, in this scenario, the coefficient of variation can be a misleading measure, because the outbreak size distribution becomes a zero-inflated bimodal (these outbreak size distributions in the workplace were previously reported in Sánchez-Taltavull et al. (2021)). Note, it is possible for a bimodal and unimodal distribution to have the same coefficient of variation (Supplementary Information, Fig. S7).

Therefore the coefficient of variation should be used with caution when community transmission is low. However, even when community transmission is low, the coefficient of variation is a reliable measure of uncertainty for large work forces.

### How Spatial Heterogeneity Determines Uncertainty Depends on Community Transmission

It has been shown previously that outbreak size depends on the level of spatial heterogeneity (Ball and Lyne 2001; Ball and Neal 2002). Additionally, spatial structure can be an effective protective measure for healthcare workers (Sánchez-Taltavull et al. 2021). We observed that how uncertainty changed with the number of groups depends on community transmission. For low community transmission, the coefficient of variation should not be used as a measure of uncertainty because it did not capture the changes observed in the outbreak size distributions. When community transmission is high the predicted uncertainty increased with the number of groups. Therefore, models accounting for spatial structure should consider uncertainty when spatial heterogeneity is high.

### Heterogeneity in Infectiousness and Susceptibility Qualitatively Differed in their Impact on Uncertainty

Previous work on how individual-level variation determines disease emergence and outbreak dynamics showed a dependence on the level of heterogeneity, but susceptibility and infectiousness are not compared in the same context (Lloyd-Smith et al. 2005; Garske and Rhodes 2008; Gomes et al. 2021, 2022). Pathogens that show heterogeneity in susceptibility can lead to similar epidemiological predictions to pathogens that show heterogeneity in infectiousness. However, we found that changing the level of overdispersion in susceptibility and infectiousness produced qualitatively different predictions for the coefficient of variation. Therefore the conclusions regarding one type of heterogeneity should not be extrapolated to the other. It should also be noted that in our study, we were interested specifically in how these types of heterogeneity influence the stochastic effects inherent in small populations. To explicitly model some susceptible individuals disproportionately interacting with asymptomatic individuals would require inclusion of explicit spatial structure, such as in a network model. Another approach would be to simulate a birth-death process in which there is overdispersion in the so-called offspring distribution of an infected individual.

### Model Limitations and Future Work

A primary limitation of this work is that we do not identify areas of the parameter space which are relevant for specific occupations (within-workplace transmission and spatial structure) or pathogens (heterogeneity in infectiousness and susceptibility). This process would allow consideration of both stochastic and parametric uncertainty. Subsequently, the model could be validated with empirical observations for various occupations. Predictions for workplace transmission of SARS-CoV-2 might be possible in this regard, as data are available on community transmission (Elson et al. 2021; Chen et al. 2021), workplace cases (Southall et al. 2021; Stringhini et al. 2021; Appleby 2021), susceptibility (Gomes et al. 2021, 2022) and infectiousness (Illingworth et al. 2021). We based our analyses around the coefficient of variation of the cumulative number of recovered individuals at the final time step. How uncertainty in transmission dynamics changes through time is also likely to be of interest (i.e. when should testing be implemented?), particularly for scenarios where a bimodal outbreak size distribution is predicted. However, although the coefficient of variation can be calculated for each time step, it does not give any information regarding the shape of the epidemic curve, only the variation across individual simulations.

## Conclusion

The detail required to accurately capture uncertainty depends on the strength of community and workplace transmission, workforce size, spatial heterogeneity and individual heterogeneity. Additionally, we have identified areas of the parameter spaces where the coefficient of variation is not a reliable measure of outbreak size uncertainty. Future mathematical models intended to inform workplace policies should carefully consider the transmission conditions and biological details of the pathogen before communicating predicted uncertainty to policy makers.

### Supplementary Information

Below is the link to the electronic supplementary material.

Supplementary file 1 (pdf 1120 KB)
