# Supplementary material for: Infectious Disease in the Workplace: Quantifying Uncertainty in Transmission
Source: Bull Math Biol. 2024 Feb 1;86(3):27. doi: 10.1007/s11538-023-01249-x (PMC10834607; doi:10.1007/s11538-023-01249-x)
Supplement: Supplementary file 1 — (pdf 1120 KB) [file 11538_2023_1249_MOESM1_ESM.pdf]

Supplementary Information

Algorithm for simulating workplace infection dynamics without accounting for spatial structure or individual variation in infectiousness or susceptibility.

Each individual, i, can be in state $S_W(i)$, $E_W(i)$, $I_W(i)$, $A_W(i)$, $R_W(i)$. To represent all individuals in each state we use S_W, E_W, I_W, A_W, R_W, that is, $\sum_i S_W(i) = S_W$, analogous for the other variables. We denote the age of infection, the duration an individual has been infected for, as s_i.

1. Set $t = 0$ and the initial conditions $(S_W, E_W, I_W, A_W, R_W) = (N_W, 0, 0, 0, 0)$, with the age of infection, $s_i = 0 \; \forall i$.
2. Calculate and store community transmission according equations 1-5 (see main text) for 110 days (dt= 4/24 days). The resulting $S_C(t), E_C(t), I_C(t), H_C(t)$ and $R_C(t)$ are used in the following steps for calculating the rate of new infections from the community (i.e. $\alpha I_C(t)$ step 3).
3. For each susceptible individual, i, we draw a value from a binomial distribution, using probability argument $1 - e^{-(\beta \frac{A_W}{N_W} + \alpha I_C(t))dt}$.
4. Each draw (i.e. individual) in the previous step giving a success (1), results in the transition $S_W(i) \rightarrow E_W(i)$.
5. For each individual moving from $S_W(i) \rightarrow E_W(i)$, assign a waiting time from an exponential distribution to remain in the exposed state, η_i.
6. For all exposed individuals where, $s_i \geq \eta_i$, we draw a value from a binomial distribution with probability argument ϵ_3.
7. Each draw in the previous step giving a success (1), results in the transition $E_W(i) \rightarrow I_W(i)$. The draws giving a failure (0), result in the transition $E_W(i) \rightarrow A_W(i)$.
8. For individuals which are selected to move to the asymptomatic class, $E_W(i) \rightarrow A_W(i)$, draw a time of recovery from an exponential distribution, ψ_i.
9. For all asymptomatic individuals, where $s_i \geq \psi_i + \eta_i$, the transition $A_W(i) \rightarrow R_W(i)$ occurs.
10. For each infected individual, we draw a value from a binomial distribution, using probability argument $1 - e^{-rdt}$.
11. Each draw in the previous step giving a success (1), results in the transition $I_W(i) \rightarrow R_W(i)$.
12. The time step is updated, $t \rightarrow t + dt$, and for all individuals in the exposed or asymptomatic classes, the age of infection is updated $s_i \rightarrow s_i + dt$.
13. if $t < 110$ days, return to step 3.

Mean field dynamics for workplace transmission

Assuming no division of the workforce or heterogeneity in infectiousness or susceptibility, the mean-field dynamics are as shown in [1]:

$$\frac{dS_W}{dt} = -\alpha(t) S_W I_C - \beta S_W \frac{A_W}{N_W} \tag{1}$$

$$\frac{dE_W}{dt} = \alpha(t) S_W I_C + \beta S_W \frac{A_W}{N_W} - l\epsilon_3 E_W - l(1 - \epsilon_3 E_W) \tag{2}$$

$$\frac{dI_W}{dt} = l\epsilon_3 E_W - rI_W \tag{3}$$

$$\frac{dA_W}{dt} = l(1 - \epsilon_3)E_W - rA_W \tag{4}$$

$$\frac{dR_W}{dt} = rA_W + rI_W \tag{5}$$

Summary for Figures S3-5

The outbreak size distribution for 15 subgroups with randomly assigned sizes is similar to that with equal group sizes, with a marginally longer tail (Fig. S3). We can also see that when we have one large group and 14 small ones, we get a qualitatively similar result to when we have 2 equally sized subgroups (a bimodal distribution, Fig. S4), this suggests that it is the size of the groups which is important, rather than the number of groups. When we make these groups more balanced, but still with one larger than the others (Fig. S5), the distance between the two modes of the distribution goes down, and we approach the same distribution produced with 15 equally sized subgroups.

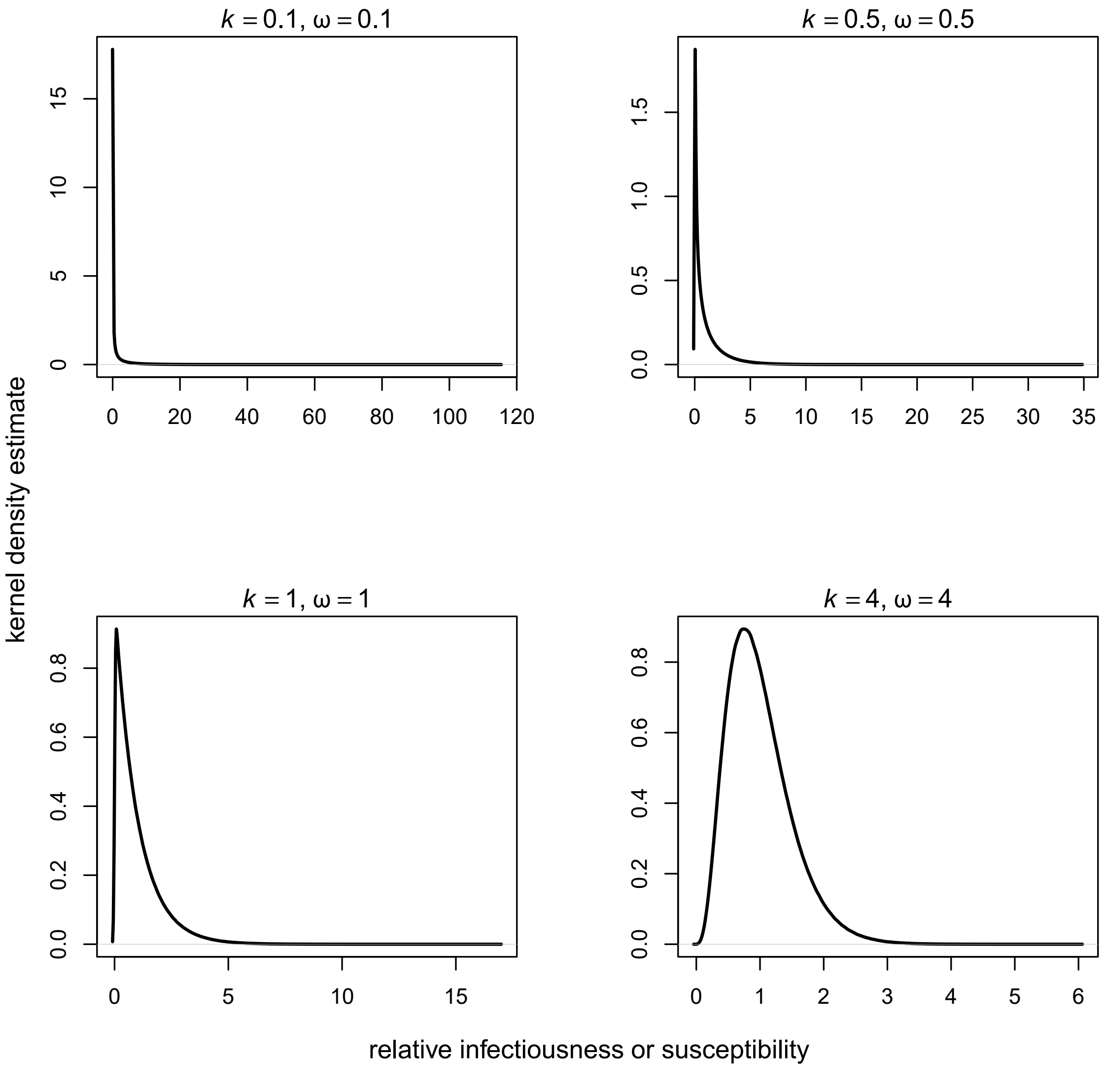

Figure S1: The gamma distribution for different values of k.

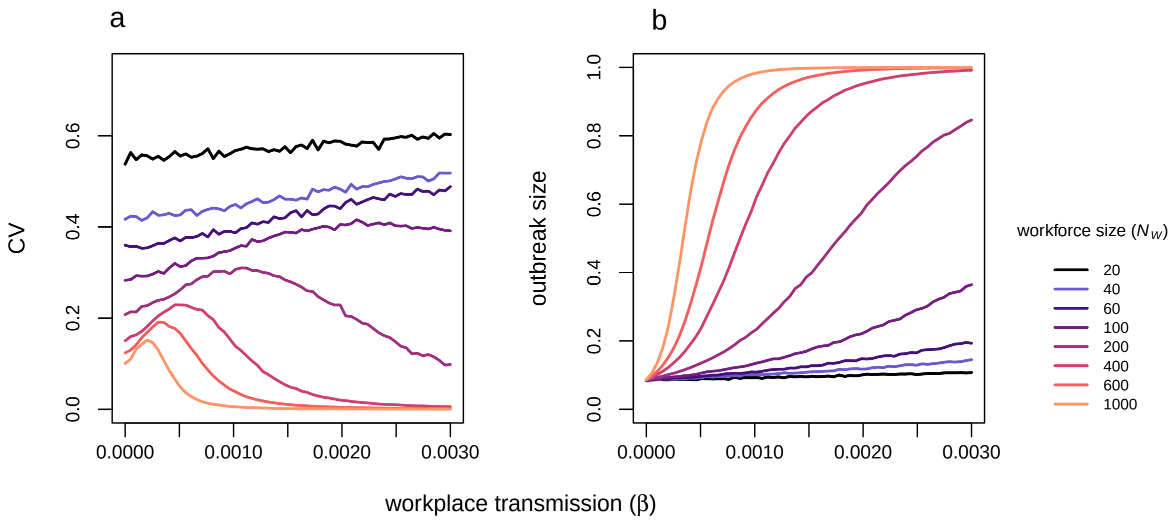

Figure S2: a The coefficient of variation for different workforce sizes and strengths of workplace transmission assuming density dependent transmission and b the mean outbreak size (cumulative number of recovered individuals at the end of the outbreak).

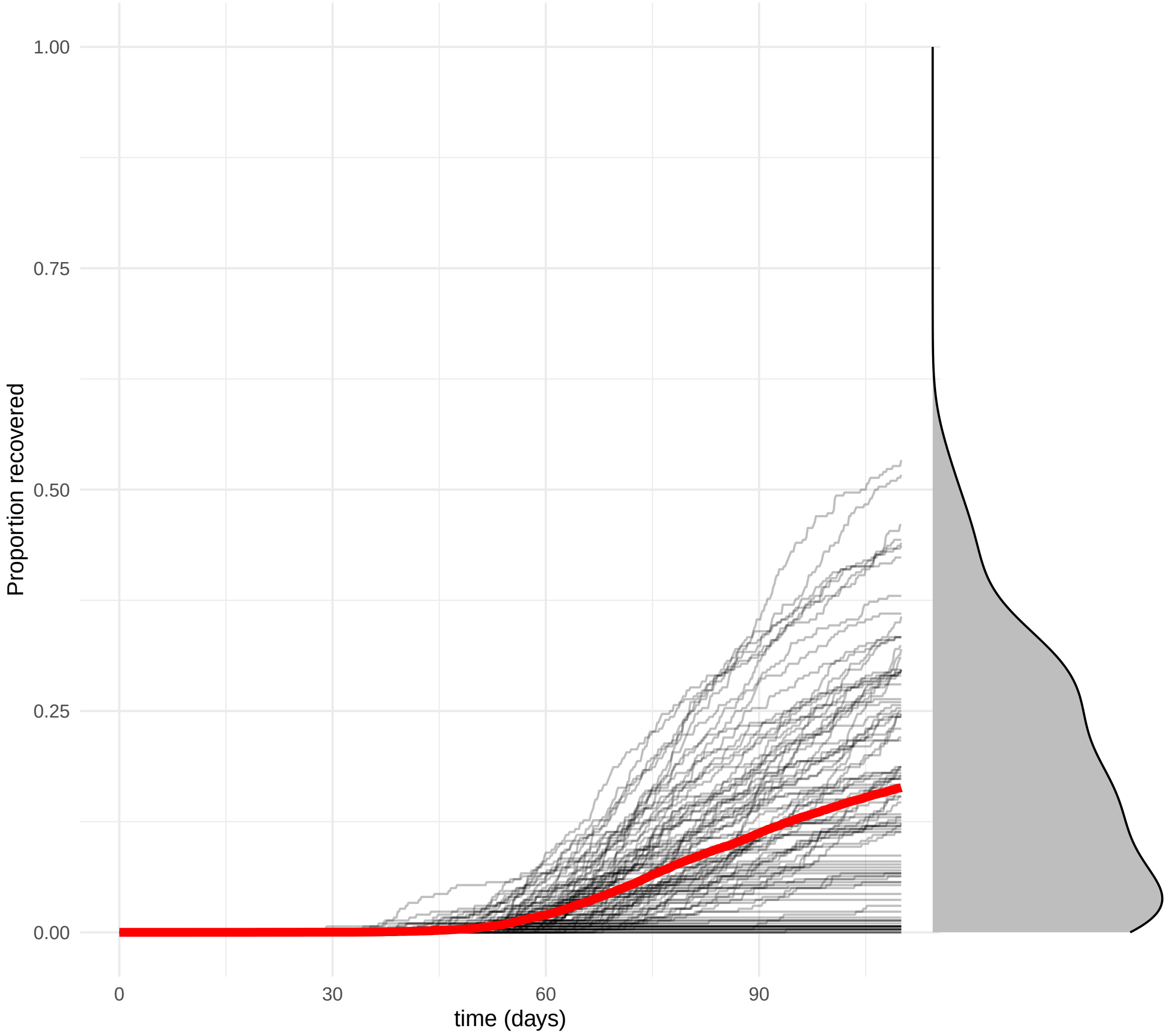

Figure S3: The temporal dynamics of the proportion of recovered individuals in the workforce and the distribution of the outbreak sizes assuming randomly assigned group sizes, for low community transmission (See Fig. 4b ii for the assumed community dynamics).

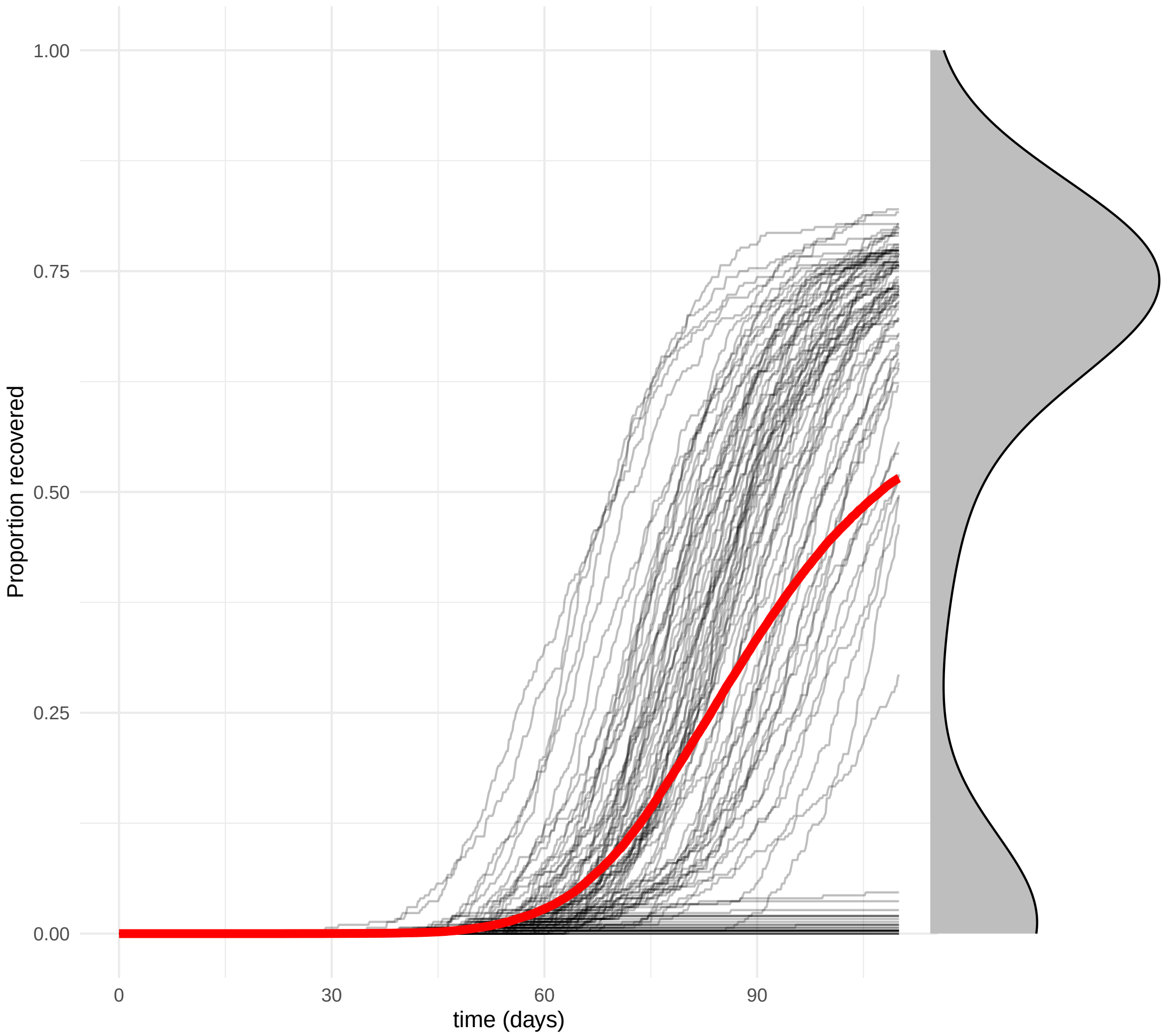

Figure S4: The temporal dynamics of the proportion of recovered individuals in the workforce and the distribution of the outbreak sizes assuming 15 subgroups with one large group and 14 small ones (1 group of 230 workers and 14 of 5 workers), for low community transmission (See Fig. 4b ii for the assumed community dynamics).

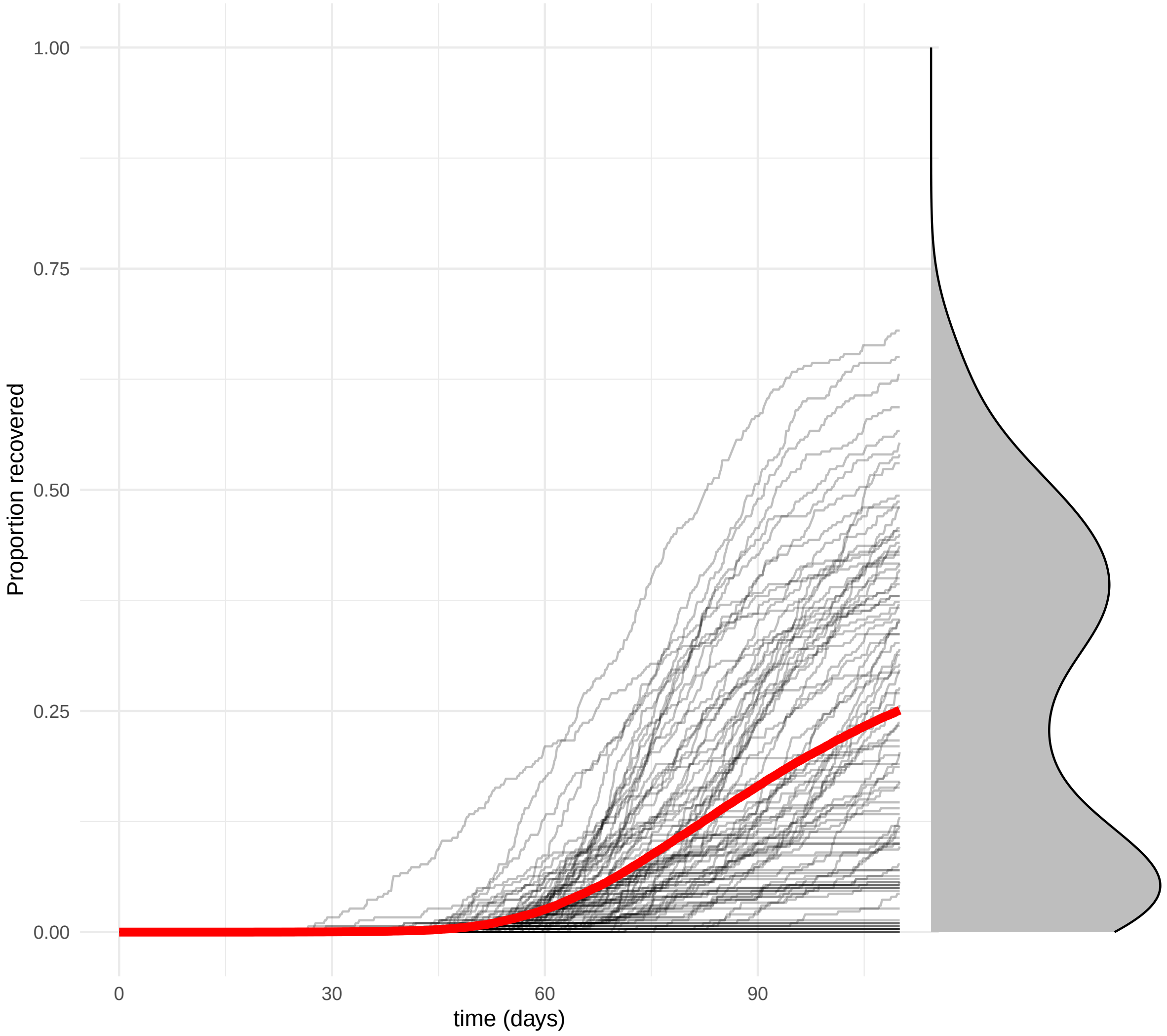

Figure S5: The temporal dynamics of the proportion of recovered individuals in the workforce and the distribution of the outbreak sizes assuming 15 subgroups with unequal group sizes but more balanced than in Fig. S4 (1 group of 90 workers and 14 of 15 workers), for low community transmission (See Fig. 4b ii for the assumed community dynamics).

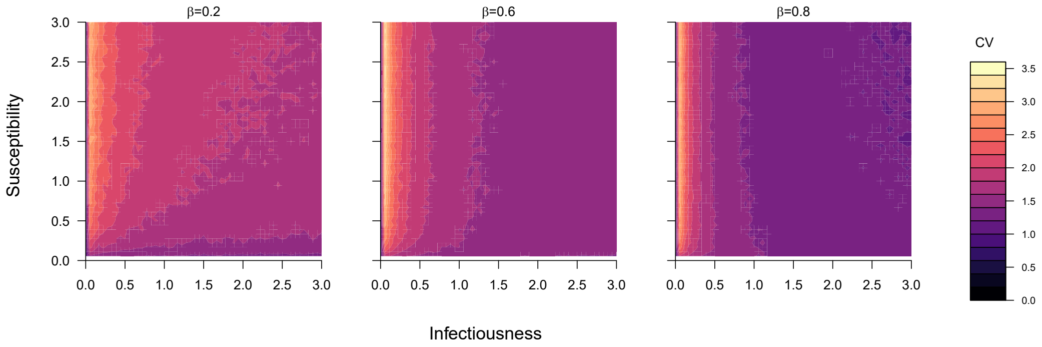

Figure S6: The coefficient of variation for outbreak size assuming both heterogeneity in infectiousness and susceptibility, for three levels of workplace transmission.

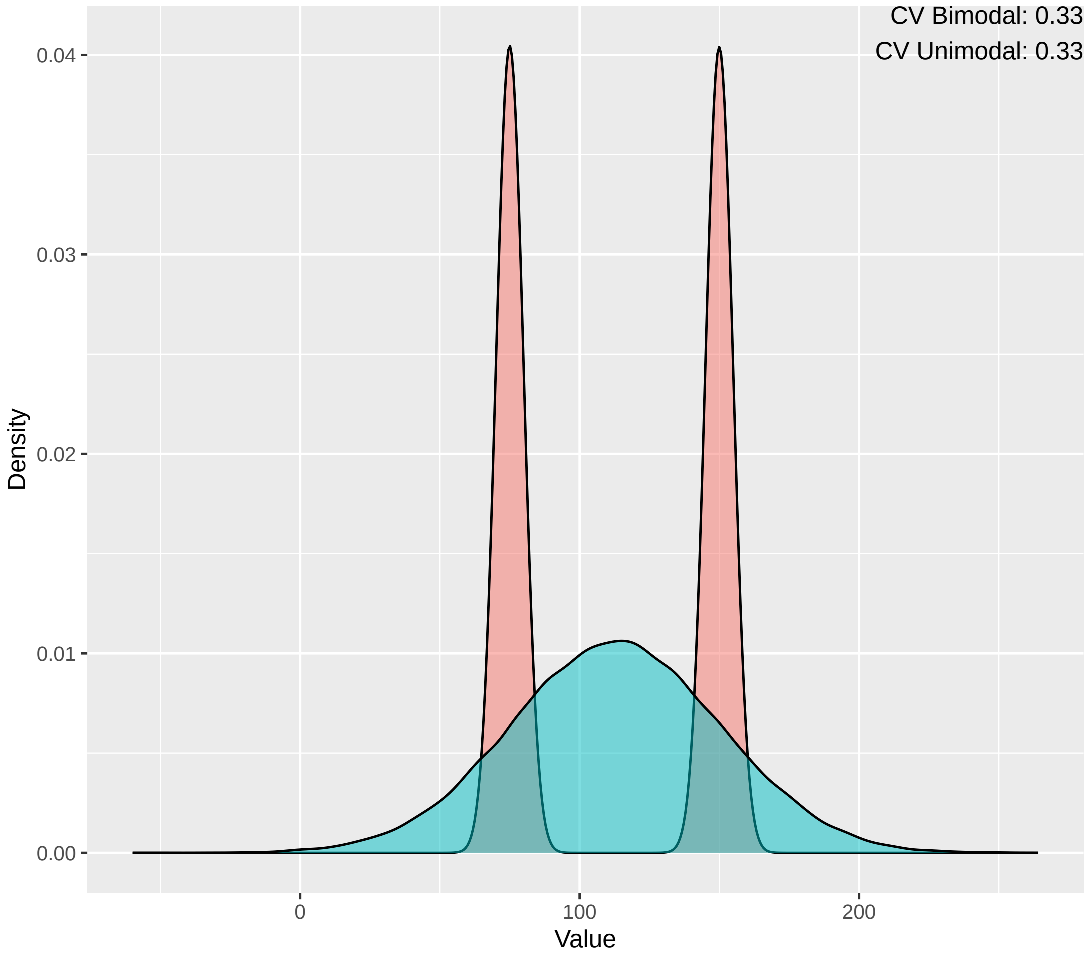

Figure S7: A unimodal and bimodal distribution with the same coefficients of variation. The coefficient of variation for both distributions is 0.33.

References

[1] Daniel Sanchez-Taltavull, Violeta Castelo-Szekely, Shaira Murugan, Jonathan ID Hamley, Tim Rollenske, Stephanie C Ganal-Vonarburg, Isabel Büchi, Adrian Keogh, Hai Li, Lilian Salm, et al. Regular testing of asymptomatic healthcare workers identifies cost-efficient SARS-CoV-2 preventive measures. *PloS One*, 16(11):e0258700, 2021.
